# FetalQuant^SD^: accurate quantification of fetal DNA fraction by shallow-depth sequencing of maternal plasma DNA

**DOI:** 10.1038/npjgenmed.2016.13

**Published:** 2016-05-11

**Authors:** Peiyong Jiang, Xianlu Peng, Xiaoxi Su, Kun Sun, Stephanie C Y Yu, Weng In Chu, Tak Y Leung, Hao Sun, Rossa W K Chiu, Yuk Ming Dennis Lo, Kwan Chee Allen Chan

**Affiliations:** 1 Centre for Research into Circulating Fetal Nucleic Acids, Li Ka Shing Institute of Health Sciences, The Chinese University of Hong Kong, Hong Kong SAR, China; 2 Department of Chemical Pathology, The Chinese University of Hong Kong, Hong Kong SAR, China; 3 Department of Obstetrics and Gynaecology, The Chinese University of Hong Kong, Prince of Wales Hospital, Hong Kong SAR, China

## Abstract

Noninvasive prenatal testing using massively parallel sequencing of maternal plasma DNA has been rapidly adopted in clinical use worldwide. Fetal DNA fraction in a maternal plasma sample is an important parameter for accurate interpretations of these tests. However, there is a lack of methods involving low-sequencing depth and yet would allow a robust and accurate determination of fetal DNA fraction in maternal plasma for all pregnancies. In this study, we have developed a new method to accurately quantify the fetal DNA fraction by analysing the maternal genotypes and sequencing data of maternal plasma DNA. Fetal DNA fraction was calculated based on the proportion of non-maternal alleles at single-nucleotide polymorphisms where the mother is homozygous. This new approach achieves a median deviation of 0.6% between predicted fetal DNA fraction and the actual fetal DNA fraction using as low as 0.03-fold sequencing coverage of the human genome. We believe that this method will further enhance the clinical interpretations of noninvasive prenatal testing using genome-wide random sequencing.

## Introduction

The discovery of circulating cell-free fetal DNA in maternal plasma^1^ has catalysed a series of new avenues for noninvasive prenatal testing (NIPT), including fetal RhD blood group genotyping,^2,3^ fetal sex determination for sex-linked disorders,^4^ chromosomal aneuploidy detection^5–10^ and diagnosis of monogenic diseases.^11–16^ The accuracy of result interpretation in these tests relies on the presence of adequate amounts of fetal DNA in a maternal sample, commonly expressed as the fetal DNA fraction. The fetal DNA fraction is directly taken into consideration in the diagnostic algorithms in many clinical applications, for example, the detection of chromosomal aneuploidies^17^ and the determination of monogenic disease inheritance.^11–16^ In particular, the fetal DNA fraction is a key parameter for determining whether the imbalance between wildtype and mutant molecules in maternal plasma is statistically significant in the diagnosis of monogenic diseases.^11,12,14^ In such analyses, fetal DNA fraction is integrated in the mathematical models that are used in the relative mutation dosage^18^ and relative haplotype dosage^12^ approaches to determine the theoretical thresholds for classifying the inherence of monogenic disorders in an unborn fetus through the analysis of maternal plasma DNA.

To date, several methods have been developed for estimating the fetal DNA fraction in a maternal plasma sample. Most of these methods are based on the quantification of fetal-specific sequences that are not present in the mother’s genome, for example, chromosome-Y sequences^5,19–21^ and paternally inherited single-nucleotide polymorphism (SNP) alleles. However, the detection of chromosome-Y sequences is only applicable for pregnancies with male fetuses. For the analyses using fetal-specific alleles, informative SNP loci where the mother is homozygous (denoted as having two A alleles, i.e., AA) and the fetus is heterozygous (denoted as AB) would be identified and the fetal DNA fraction is calculated based on the ratio of the paternally inherited fetal allele (B allele) and the allele shared between the mother and the fetus (A allele). Most of these methods would require the genotypic information of both parents to identify the informative SNP loci.^12,22^ The acquisition of paternal genotype could present practical difficulties because (a) only maternal blood samples would be collected for prenatal testing in most clinical settings and (b) the paternal genotype information may not be accurate due to non-paternity.^23^ In this regard, we have previously developed a method that does not require prior parental genotype information.^24^ In that method, SNP loci showing two different alleles in plasma are first identified. Bayesian statistical analysis is applied to determine the fetal DNA fraction based on the ratios of the two alleles.^24^ As this approach requires a sequencing depth of over 120× to ensure that the fetal-specific allele can be detected, targeted sequencing, for example, through the use of hybridization- or amplicon-based enrichment systems, would be required.^17,22^

NIPT for chromosomal aneuploidies has been rapidly adopted for clinical service in >90 countries globally.^25,26^ One widely used approach for performing NIPT for aneuploidies is random sequencing of plasma DNA of pregnant women.^7^ In this approach, maternal plasma DNA is randomly sequenced with a relatively low-sequencing depth and mapped to a reference genome. An aneuploid chromosome would lead to an increased or reduced representation of the chromosome in the maternal plasma. For example, a trisomy 21 fetus would release an increased amount of chromosome 21 sequences into maternal plasma. Using this approach, the overall detection rate of trisomy 13, 18 and 21 was reported to be 98.9% with ~0.2-fold sequencing coverage of the human genome.^27^ It would be useful if the fetal DNA fraction can be accurately and robustly determined in maternal plasma samples subjected to shallow-depth random sequencing. In this regard, size-based^10^ and methylation-based^28,29^ methods have been developed. However, these methods are generally less precise and accurate than methods based on fetal-specific alleles for the estimation of fetal DNA fraction. Recently, a new approach for fetal DNA fraction estimation based on random sequencing of the maternal plasma has been developed through analysing the tag densities within different windows,^30^ however, this approach might have reduced robustness for measuring the fetal DNA fraction <5%.^30,31^

In this study, we proposed a new method for determining fetal DNA fraction in maternal plasma by sequencing the maternal plasma DNA at a shallow depth, for example, 0.03-fold coverage of the human genome. This sequencing depth is readily achievable for most of the routine clinical service for NIPT of chromosomal aneuploidies. Therefore, this new method can be easily adapted to the protocols currently used by laboratories offering NIPT service. The resultant availability of accurate fetal DNA fraction information would be useful for quality control and might be incorporated into the diagnostic algorithms to improve diagnostic performance.

This new method was named as FetalQuant^SD^. ‘SD’ stands for ‘shallow depth’ of sequencing data, which was used to highlight the difference from our previous algorithm ‘FetalQuant’^24^ that uses high-depth sequencing data (e.g., targeted sequencing).

## Results

### Principle

**Figure 1:**
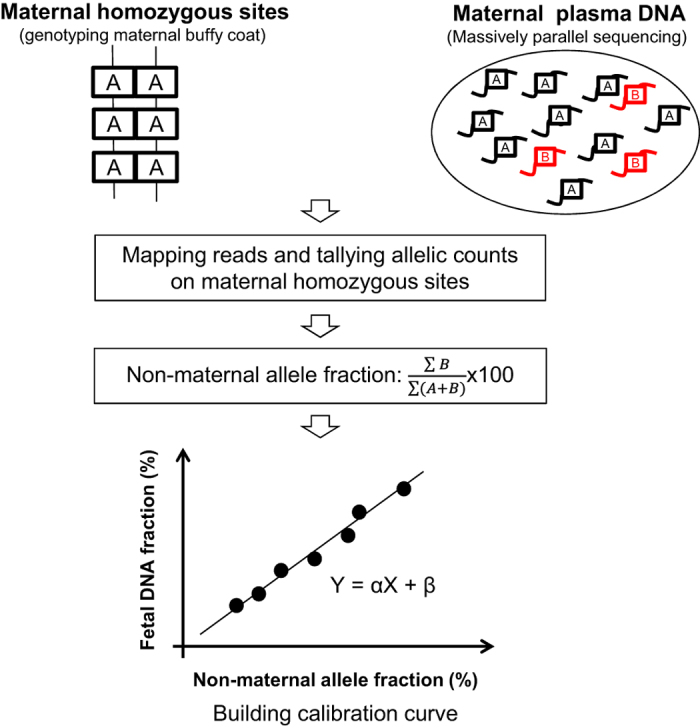
Schematic illustration of the principle used in FetalQuant^SD^. Sequence reads were aligned to the human reference genome and compared with the sites where the maternal genotypes were homozygous. The non-maternal allele fraction can be inferred by aggregating all the reads carrying an allele different from the corresponding maternal allele across the maternal homozygous sites in a genome-wide manner.

The principle of this method is illustrated in Figure 1. Briefly, maternal blood cells were genotyped using microarray-based genotyping technologies to identify SNP loci where the pregnant woman is homozygous. Then, sequenced reads with non-maternal alleles were identified from the maternal plasma DNA-sequencing results. These non-maternal alleles would potentially represent paternally inherited fetal alleles. However, a small proportion of these non-maternal alleles could be caused by sequencing errors in maternal plasma and/or genotyping errors in maternal genomic DNA. Assuming that the error rates are relatively constant across different cases, the fetal DNA fraction would be proportional to the fraction of non-maternal alleles measured in maternal plasma.

### Correlation between fetal DNA fraction and the proportion of non-maternal alleles in maternal plasma

**Figure 2:**
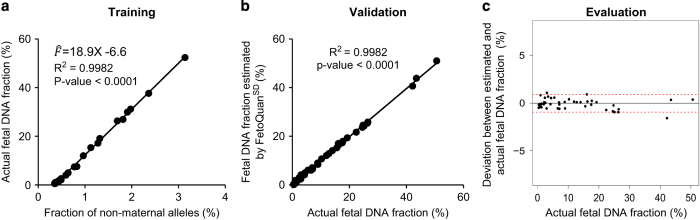
Linear regression model construction and validation. The regression model was constructed using the training data set (**a**) and validated in an independent data set (**b**). The deviations between the estimated and actual fetal DNA fraction were shown in **c**. Horizontal red lines represent the 95% confidence interval of deviations.

A linear relationship was observed between the proportion of non-maternal alleles in maternal plasma and the actual fetal DNA fraction for the training data set consisting of 23 samples (*R*^2^=0.99 and *P*<0.0001, linear regression, Figure 2a). Thereby, we built a linear regression model to describe how the actual fetal DNA fraction is correlated with the proportion of non-maternal alleles in the maternal plasma, deriving the following equation:

$$\hat{F}=18.9X-6.6$$

where $\hat{F}$ is the estimated fetal DNA fraction and *X* is the percentage of non-maternal alleles in the plasma sample. To evaluate the accuracy of this regression model, we further applied it to an independent validation data set.

### Accuracy of fetal DNA fraction estimation

The estimated fetal DNA fractions correlated well with the actual fetal DNA fractions (*R*^2^=0.99, *P*<0.0001, linear regression, Figure 2b). The median of absolute deviation from the actual fetal DNA fraction was 0.4% (range: −1.6 to 1.1%, Figure 2c). The 95% confidence interval for the deviation was from −0.95 to 0.9% (Figure 2c).

### Fivefold cross-validation analysis

**Figure 3:**
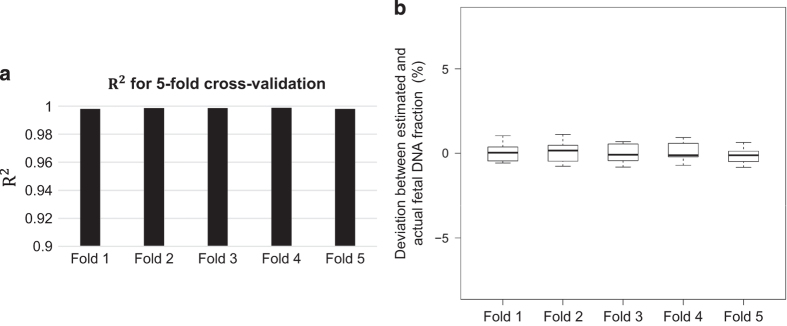
Fivefold cross-validation analysis. (**a**) *R*^2^ of linear regression at each fold. (**b**) Boxplot of deviations between the estimated and actual fetal DNA fraction at each fold.

Fivefold cross-validation analysis was conducted to demonstrate the robustness of the linear regression model deduced in this study. The mean values of the slopes and intercepts for these linear regression models across the fivefold cross-validation results are 19.0 (range: 18.879–19.063) and 6.62 (range: 6.53–6.68), respectively, which is close to the aforementioned linear model ($\hat{F}=18.9X-6.6$) deduced from 23 samples. Moreover, the mean of *R*^2^ values across all folds was 0.998 (Figure 3a) and the mean value of the absolute deviations is 0.5% (Figure 3b), suggesting that this linear regression model could be well reproduced in the independent validation subsets.

### Factors affecting the accuracy

#### Sequencing depth

**Figure 4:**
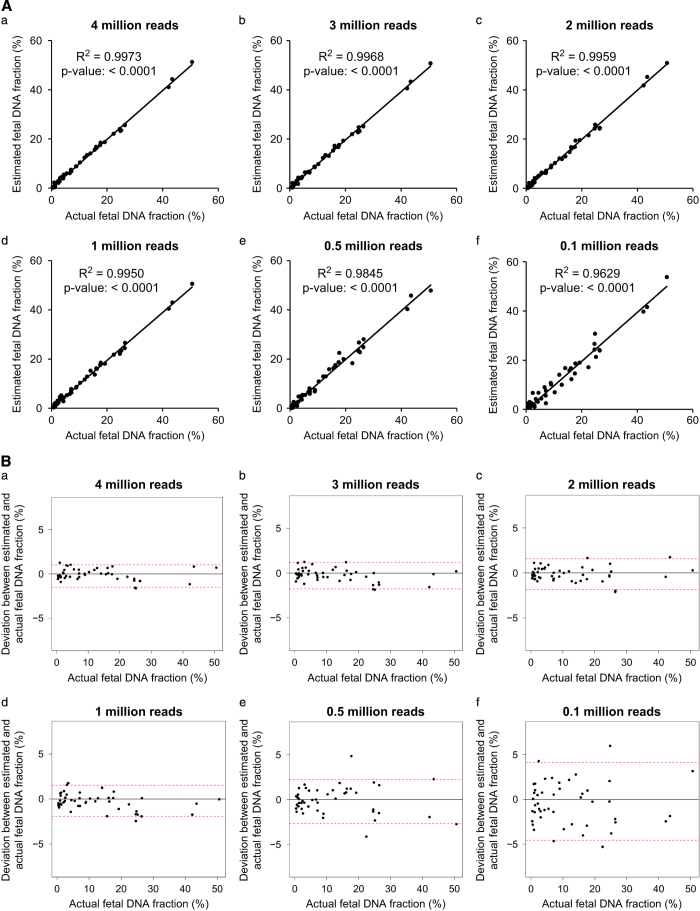
Evaluation on the effect of sequencing depth on estimated fetal DNA fraction. (**A**) Comparison between the estimated and actual fetal DNA fraction at different sequencing depths. (**B**) Deviations between the estimated and actual fetal DNA fraction at different sequencing depths. Horizontal red lines represent the 95% confidence interval of deviations.

To further demonstrate how sequencing depth would affect the measured fetal DNA fraction, we performed downsampling analysis. We randomly selected paired-end reads of 4, 3, 2, 1, 0.5 and 0.1 million per sample independently each time from the original sequencing data in the validation data set and repeated the aforementioned fetal DNA fraction prediction. As a result, for data using 1 million reads, the median of the absolute deviation reaches 0.61% (95% confidence interval: −1.93% to 1.52%.), which visually provides a similar performance compared with that of 4 million reads (Figure 4).

#### The number of SNPs

**Figure 5:**
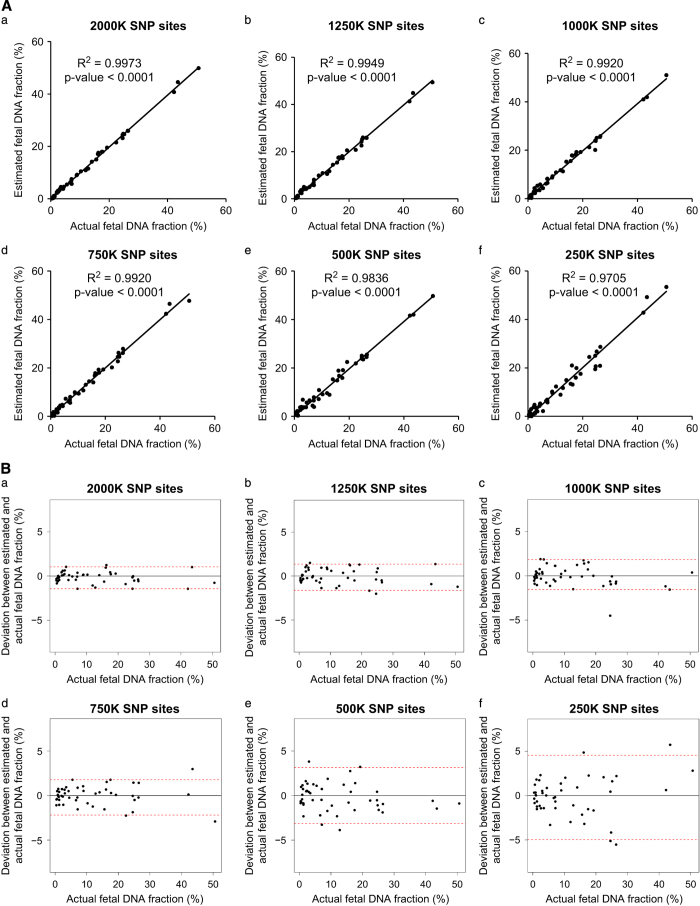
Evaluation on the effect of SNP count on estimated fetal DNA fraction. (**A**) Comparison between the estimated and actual fetal DNA fraction at different number of SNPs. (**B**) Deviation between the estimated and actual fetal DNA fraction at different number of SNPs. Horizontal red lines represent the 95% confidence interval of deviations.

We further explored how the number of SNPs would affect the accuracy of fetal DNA fraction estimation when 1 million reads were used. Thus, 2,000, 1,250, 1,000, 750, 500 and 250 K SNPs were randomly selected from the full data set. A total of 750 K SNPs were sufficient to give an accurate prediction showing a median deviation of 0.52% (95% confidence interval: −2.19 to 1.77%; Figure 5).

#### Impact of sequencing depth and the number of SNPs upon the accuracy

**Figure 6:**
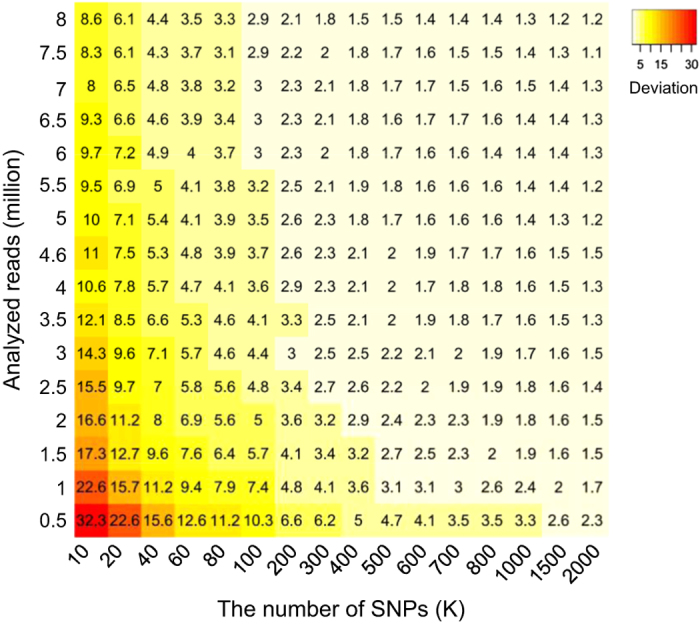
Impact of sequencing depth and the number of SNPs on the accuracy of fetal DNA fraction estimation. The number in each grid indicates half the width of the 95% confidence interval of deviations at a given sequencing depth and number of SNPs. The gradient colour on each grid of the heat map denotes the performance of each combination.

Because both the number of SNPs and sequencing depth would influence the accuracy of fetal DNA fraction measurement, we repeated the aforementioned simulation analyses to investigate the accuracies corresponding to combinations of different number of SNPs and sequencing depths. Figure 6 shows the deviation at 95% confidence interval at a given number of SNPs and a particular sequencing depth. For example, 8 million reads and 300 K SNPs could give a deviation of ±1.8%.

## Discussion

The accurate interpretation of the result of NIPT is affected by the amount of fetal DNA in the maternal plasma sample. Thus, the accurate measurement of the fetal DNA fraction is crucial for such testing. In this study, we have developed a new methodology to estimate the fetal DNA fraction, leveraging on the relationship between the fetal DNA fraction and the fraction of non-maternal alleles present in the plasma of a pregnant woman. Good linearity between the actual fetal DNA fraction and the fraction of non-maternal alleles was observed in the maternal plasma. These results demonstrated that the genotyping and sequencing errors were relatively constant in our hands. The predictive ability of this method has been validated in an independent data set.

Notably, the sequencing depth allowing an accurate fetal DNA fraction estimation can be as low as 0.03-fold human-genome coverage as demonstrated in the downsampling analysis. Our results suggested that this method could be robustly applied to samples undergoing NIPT for chromosomal aneuploidy detection using shallow-depth random sequencing.^27,30^ The number of SNPs required can be as little as 300,000 when 8 million reads are used (Figure 6). The small number of SNPs required can allow us to perform multiplex genotyping of maternal buffy coat samples to get maternal genotype information in a cost-effective manner. For example, the HumanCore-24 v1.0 DNA Analysis Kit (Illumina, San Diego, CA, USA) is capable of genotyping 48 samples, each for 300,000 SNPs, at a cost of USD 2,400. In other words, it would cost an extra USD 50 for each sample to obtain the maternal genotype information for fetal DNA fraction estimation. According to the simulation, 300,000 SNPs would achieve an accurate fetal DNA fraction as suggested by the deviation of ±1.8% (Figure 6). The sample throughput of genotyping is greater than 2,800 per week. Therefore, we believe that this method should be practicable in actual clinical use.

The accuracy of the fetal DNA prediction using this method should be higher than two previous non-polymorphism-based approaches.^10,30^ The reported correlation coefficients between the measured and the actual fetal DNA fraction were 0.83 and 0.93 in these previous studies^10,30^, whereas the value in our approach is 0.99. Furthermore, the new method, FetalQuant^SD^, can accurately measure the fetal DNA fraction of even below 5% as suggested by the median deviation of 0.6% (95% confidence interval: −1.2 to 1.7%) using 1 million reads (Figure 4B). This ability to measure low fetal DNA fractions is particularly important because the accurate estimation of fetal DNA fraction will allow us to identify samples with low fetal DNA fractions so as to reduce the chance of false-negative results.^5,27^

However, with different sequencing and genotyping platforms used, the training process would need to be repeated to deduce a new set of parameters for the linear regression model. In addition, there are two extra factors that might affect the fetal DNA fraction estimation, namely the uneven GC content and allele-specific copy-number variations. However, even though the uneven GC content across the human genome has been reported to affect the quantification of sequence reads across different genomic regions,^32^ the allelic ratio metric used in this study would control for the GC bias because two quantifications derived from the same region are compared. As the frequency of copy-number variations^33^ and SNPs^34^ in the human genome is relatively low, the specific bias caused by certain copy-number variations would contribute little to the current linear regression model.

With the rapidly reducing cost and increasing accessibility of personal genome-sequencing analysis,^35^ individual genotype information will become more readily available in the near future. In the event that a pregnant subject already has genotyping information generated from a previous analysis, the maternal genotype-assisted fetal DNA fraction estimation would be readily integrated into currently existing approaches used in NIPT without any additional cost. Therefore, this method would serve as an accurate and robust method for quality control for NIPT and may lead to algorithms for improved diagnostic performance.

## Materials and methods

### Subjects

In our previous study regarding high-resolution profiling of fetal DNA clearance from maternal plasma by massively parallel sequencing,^36^ we collect predelivery and serial postdelivery maternal plasma. As a result, 70 plasma samples from our previous study,^36^ which were recruited with informed consent from 12 women with uncomplicated singleton pregnancies were analyzed in this study. The blood cells were genotyped using the BeadChip array (Illumina) and the maternal plasma DNA samples were sequenced using the HiSeq 2000 platform (Illumina) with a 50-cycle paired-end mode.^36^ On an average, 1.94 million (range: 1.92 to 1.95 million) homozygous loci were obtained for each case for the 2.35 million SNPs on the BeadChip array. A median of 139.9 million (range: 44 to 188 million) alignable nonduplicated reads were obtained for each plasma sample.

To evaluate the performance of the fetal DNA fraction prediction, the estimated fetal fraction was compared with the fetal DNA fraction that was determined through the use of the maternal and fetal genotypes as the gold standard (deemed as the actual fetal DNA fraction) according to the study by Lo *et al.*^12^

### Calculation of fetal DNA fraction

The samples were randomly divided into a training set (23 samples) and an independent validation set (47 samples). Linear regression was performed to determine the relationship between the actual fetal DNA fraction and the fraction of non-maternal alleles in maternal plasma. The actual fetal DNA fraction (*F*, deemed as the gold standard) was deduced by comparing the aligned sequence reads to the sites where the maternal genotypes were homozygous (AA) and the fetal genotypes were heterozygous (AB) using the following formula.^12^

$$F=\frac{2p}{p+q}\times 100$$

where *p* is the number of sequenced reads carrying fetal-specific alleles (i.e., allele B) and *q* is the number of sequenced reads carrying alleles shared by the mother and the fetus (i.e., allele A). The fraction of non-maternal alleles were calculated by comparing the aligned sequence reads to the sites where maternal genotypes were homozygous but fetal genotypes were not required to be known.

### Fivefold cross-validation

Seventy samples were randomly partitioned into 5 equal-size subsets, with 14 samples in each subset. Fivefold cross-validation was performed in such a way that a single subset was retained as the validation data for testing the model, whereas the remaining subsets were used as the training data to construct the linear regression model regarding the relationship between the actual fetal DNA fraction and non-maternal allele fraction. The cross-validation process was repeated five times (also referred to as folds). Then *R*^2^ values and deviations from the fivefold cross-validation results were used to evaluate the robustness of the deduced linear regression model.

### Computational simulation for studying the impact of sequencing depth and the number of SNPs upon the accuracy

Sixty five samples with over 80 million reads each were used to study how sequencing depth and the number of SNPs would impact upon the accuracy of fetal DNA fraction estimation through the use of downsampling analysis (Figure 6). For each combination of a given SNP count and sequencing depth, the 95% confidence interval for deviations between the estimated and actual fetal DNA fraction were calculated according to the downsampling analysis by 20 times. Then, we used a heat map to graphically visualise half the width of the 95% confidence interval in order to demonstrate the influence of sequencing depth and the number of SNPs on the accuracy of fetal DNA fraction estimation.

### Implementation of FetalQuant^SD^

FetalQuant^SD^ was implemented using Perl (https://www.perl.org/) and R (https://www.r-project.org/) languages. It was designed to run on a x86_64 GNU/Linux platform. Perl script was used to calculate the allelic ratio and R language was used to construct the linear regression model. The source code was available at http://www.cuhk.edu.hk/med/cpy/Research/FetalQuantSD/.

### Availability of data and materials

The sequence data for the subjects studied in this work who have consented to data archiving have been deposited in the European Genome-Phenome Archive (EGA), www.ebi.ac.uk/ega, hosted by the European Bioinformatics Institute (EBI), www.ebi.ac.uk (accession no. EGAS00001001611).
